# Biocompatibility and sub-chronic toxicity studies of phlorotannin/polycaprolactone coated trachea tube for advancing medical device applications

**DOI:** 10.1038/s41598-024-54684-8

**Published:** 2024-02-16

**Authors:** Tae-Hee Kim, Seong-Yeong Heo, Gun-Woo Oh, Won Sun Park, Won-Kyo Jung

**Affiliations:** 1 Research Center for Marine-Integrated Bionics Technology, Pukyong National University, Busan, 48513 Republic of Korea; 2 Marine Integrated Biomedical Technology Center, The National Key Research Institutes in Universities, Pukyong National University, Busan, 48513 Republic of Korea; 3 Jeju Bio Research Center, Korea Institute of Ocean Science and Technology (KIOST), Jeju, 63349 Republic of Korea; 4 National Marine Biodiversity Institute of Korea, Seochun, Chungcheongnam-do 33662 Republic of Korea; 5 Department of Physiology, Kangwon National University School of Medicine, Chuncheon, 24341 Republic of Korea; 6 Major of Biomedical Engineering, Division of Smart Healthcare, College of Information Technology and Convergence and New-Senior Healthcare Innovation Center (BK21 Plus), Busan, 48513 Republic of Korea

**Keywords:** Biocompatibility, Sub-chronic toxicity, Endotracheal tube, Phlorotannin, Medical device, Biochemistry, Biotechnology

## Abstract

The phlorotannin-polycaprolactone-coated endotracheal tube (PP tube) has been developed with the aim of preventing tracheal stenosis that can result from endotracheal intubation, a factor that can lead to a serious airway obstruction. Its preventive efficacy has been assessed through both in vitro and in vivo investigations. However, there is a lack of studies concerning its biocompatibility and sub-chronic toxicity in animal models, a crucial factor to ensure the safety of its usage as a functional endotracheal tube. Thus, this study aimed to evaluate the biocompatibility and sub-chronic (13 weeks) toxicity of the PP tube through L929 cell line and diverse in vivo models. The cytotoxicity testing was performed using the extracts of PP tube on L929 cells for 72 h. Furthermore, other tests conducted on animal models, including ICR mice (acute systemic toxicity), New Zealand white rabbit (intradermal reactivity and pyrogen tests), guinea pig (maximization sensitization), and Sprague Dawley rats (sub-chronic toxicity). In both biocompatibility and sub-chronic toxicity analyses, no significant adverse effects are observed in the groups exposed to the PP tube, when compared to control group. Altogether, the findings suggested that the PP tube exhibits relative non-toxic and safety, supporting its suitability for clinical usage. However, extended periods of intubation may produce mild irritant responses, highlighting the clinical caution of limiting intubation duration to less than 13 weeks.

## Introduction

Tracheal stenosis (TS), a serious respiratory disease, is mainly resulted from prolonged endotracheal intubation and progressed through initial mucosal injury, ensuing inflammation, and fibrogenesis^1^. It presents with the proliferation of airway granulation tissue and development of fibrous scar, ultimately leading to the progressive narrowing of the tracheal lumen^2^. Current treatment modalities for TS contain endoscopic techniques, such as balloon dilatation or airway stents, for mild cases, and surgical interventions, like cricotracheal reconstruction or tracheal resection with end-to-end anastomosis for severe cases. However, these approaches frequently yield inconsistent and unsatisfactory outcomes, occasionally necessitating secondary surgery and stent placement in severe cases^3,4^. Therefore, there is an imperative need to enhance the functionality of endotracheal tubes and investigate more consistent and effective strategies for managing TS with minimal side effects.

Previous studies have reported that the excessive inflammatory response and accumulation of extracellular matrix at the injured site contribute to abnormal tissue regeneration^5,6^. Therefore, many researchers have focused on developing various materials for modifying the surfaces of endotracheal tubes or stents to prevent stenosis, excessive inflammatory response, or granulation^7–9^. For this reason, we investigated an endotracheal tube integrated with an anti-inflammatory and anti-fibrotic agent, presenting a potentially therapeutic approaches for TS.

In previous studies, phlorotannin from *Ecklonia cava*, a distinct group of polyphenolic compounds extracted from brown algae, has demonstrated numerous biological effects, including anti-inflammatory, anti-diabetic, anti-fibrosis, and anti-adhesion activities^10–13^. Building upon these findings and aiming to overcome the limitations of treatments for TS, we developed a polycapolactone-coated endotracheal tube incorporating phlorotannin (PP tube), which possesses anti-inflammatory and anti-fibrogenic properties^14^. Additionally, we investigated the active compounds in phlorotannin for its anti-fibrosis effect and underlying mechanisms^15,16^.

However, to the best of our knowledge, no study has reported on the biocompatibility and sub-chronic toxicity of the PP tube using both in vitro and in vivo models. As medical devices may release compounds or possess surface properties that could potentially induce undesirable toxicity upon clinical application, it is essential to assess and establish the biocompatibility and safety of medical devices intended for human use^17^. Through biocompatibility and safety evaluations of medical devices, both in preclinical and clinical studies, are mandated by regulatory guidance, including US Food and Drug Administration (FDA) and the International Organization for Standardization (ISO), as part of the regulatory approval process. Hence, sufficient biocompatibility and safety assessments are warranted to ensure the medical application of the PP tube in accordance with the regulatory requirements of the FDA and ISO. Our study aimed to evaluate the biocompatibility and sub-chronic toxicity of the PP tube of accordance with ISO standards, achieved through the administration of its extracts and implantation in the gluteal muscle.

## Materials and methods

### Overall design of the toxicology program

The biocompatibility testing, including cytotoxicity test, acute systemic toxicity test, pyrogen test, intradermal reactivity test, and maximization sensitization test, and sub-chronic toxicity testing were conducted by Korea Conformity Laboratories (KCL) (Geumcheon-gu, Seoul, Republic of Korea). The experiment complied with the nonclinical test management standards from the Ministry of Food and Drug Safety (MFDS) and the Organization for Economic Cooperation and Development (OECD) Principle of Good Laboratory Practice. The study was reviewed and assessed following the regulations for the Institutional Animal Care and Use Committee (IACUC) at KCL (ANI/032).

### Materials

The fabrication of the PP tube followed the procedure described by Lee et al.^14^. Briefly, a LEVIN-tube (16 French, Sewoon Medical, Cheonan, Republic of Korea) was used for the fabrication of the endotracheal tube. Polycaprolactone (PCL) pellets (5 g) were dissolved in dichloromethane (50 mL), and phlorotannin (2 g) was dissolved in 70% EtOH (40 mL). The LEVIN-tube was coated with the PCL and phlorotannin solution at room temperature for 24 h. Subsequently, the fabricated tube was washed five times with distilled water and dried at room temperature overnight. Finally, we obtained the PP-tube, composed of 10% PCL and 5% phlorotannin. The PP tube was stored at 4 °C for further experiments.

### Cell culture

The L929 cell line (murine fibroblast, passage 10–16) was obtained from the American Type of Culture Collection (ATCC, Manassas, VA). These cells were cultured in minimum essential medium (MEM) supplemented with 10% fetal bovine serum. Incubation was performed at 37 °C under a humidified atmosphere with 5% CO_2_, and sub-culturing was performed upon reaching a confluence of 70–80%.

### Experimental animals and housing conditions

Throughout the experiment, the animals were identified by numbers tattooed on the auricle. Individual identification cards of different colors were attached to the breeding box, and identification records were displayed on the door of each animal accommodation. The room temperature was maintained at 22 ± 3 ℃ with a relative humidity of 50 ± 20%. The air recycling rate was set at 10–15 times/h, and the light intensity was adjusted 150–300 Lux with a 12-h light/dark cycle, allowing free access to food and water. Throughout the entire study period, temperature and relative humidity were automatically monitored every half-hour using automatic instruments, and the environmental conditions were periodically measured. The number of animals used in our study is indicated in Supplementary Table 1. All animal treatments and surgical procedures were performed according to the guidelines for the Animal Care and Use Committee and were approved by the Animal Care and Use Committee of KCL. Every effort was made to minimize the pain and suffering of the animals.

### Biocompatibility testing

The biocompatibility testing was conducted using extracts of PP tube in MEM, saline, or cottonseed oil (CSO) as the test group, while extraction vehicles were used as negative control (NC). In particular, for the in vitro cytotoxicity assessment, 0.1% ZDEC polyurethane film and high-density polyethylene film were used as positive reference material (RM) and negative RM, respectively (Supplementary Table 2).

#### In vitro cytotoxicity test

To evaluate the in vitro cytotoxicity of PP tube, 0.1% ZDEC polyurethane film (PF), and high-density polyethylene film (HDPF) were prepared according to ISO 10993–12:2014 (Biological evaluation of medical devices-Sample preparation and reference materials) for the in vitro experiments^10^. Briefly, 120 cm^2^ of PF and HDPF were immersed in 20 mL MEM and incubated at 37 °C for 24 h. For the PP tube, 4 g of the PP tube were placed in 20 mL MEM and then incubated at 37 °C for 24 h.

L929 cells were seeded in 6-well plates at a density of 2 × 10^5^ cells/well in 2 mL of MEM. On the following day, the cells were exposed to various extracts and incubated for an additional 48 h. Subsequently, the L929 cells were washed with PBS and detached using 0.25% Trypsin–EDTA. After centrifugation at 1250 rpm for 3 min to obtain the cell pellet, the cell pellet was resuspended for counting using a hematocytometer. The data are expressed as the mean percentage of viable cells ± standard deviation (S.D.) of triplicate experiments.

#### Acute systemic toxicity study

The in vivo acute systemic toxicity analysis of the PP tube were performed by administration of extracts of the PP tube, either in saline or CSO, via injections into mice. This assessment was conducted according to ISO 10993-11:2017(E): Tests for systemic toxicity-Acute systemic toxicity^18^. ICR mice were provided by Koatech Co. Ltd. (Pyeongtaek, Gyeonggido, Republic of Korea). Twenty female mice, aged 5 weeks and weighing 17–23 g at the beginning of the dose injection, were used in acute systemic toxicity study.

The PP tube extracts were obtained by immersing in extraction vehicles (saline or CSO, respectively) and allowing to soak at 37 °C for 72 h. After preparation, the extracts were administered via two different routes: intravenous (I.V.) injection (extracts of PP tube in saline and extraction vehicle) and intraperitoneal (I.P.) injection (extracts of PP tube in CSO and extraction vehicle). The injection volume of the extracts for each animal was 50 mL/kg according to ISO standards. To evaluate acute systemic toxicity, clinical symptoms and abnormalities in appearance were observed during the experiment period, and body weight was measured 24 and 48 h after injection.

#### Intradermal reactivity test

The intradermal reactivity evaluation of the PP tube was conducted by injecting the PP tube extracts into New Zealand white rabbits. This assessment was performed following the protocol outlined in ISO 10993-10-2010(E): Tests for irritation and skin sensitization-Animal intracutanous (intradermal) reactivity test^19^. The New Zealand white male rabbits were provided by PiZhou Dongfang Rabbit Breeding Co., Ltd (PiZhou, China). The extracts of the PP tube in saline or CSO were prepared in a similar manner to that described above (2.5.2. Acute systemic toxicity study). Three New Zealand white rabbits, aged 11 weeks and weighing over 2 kg at beginning of the dose injection, were used in the intradermal reactivity test.

The dorsal surface hair of the rabbits was shaved before 4–18 h of injection. Subsequently, the animals were subjected to intracutaneous injection at a volume of 0.2 mL/injection site, as shown in Supplementary Fig. 1. Injection sites were inspected immediately post-injection and again 24, 48, and 72 h post-injection. For the assessment and scoring of outcomes such as erythema, eschar formation, and edema, a grading standard was described in Supplementary Table 3. The scores were expressed as the cumulative sum of scores for erythema and eschar formation and edema across fifteen sties divided by 15 for calculation.

#### Pyrogen test

The rabbit pyrogen test was performed by injecting extracts of PP tube in saline into New Zealand white rabbits. This assessment followed the guidelines specified in ISO 10993-11:2006(E): Tests for systemic toxicity-Annex(F) information on material mediated pyrogens^20^. New Zealand white male rabbits were provided by PiZhou Dongfang Rabbit Breeding Co., Ltd (PiZhou, China). The extracts of PP tube in saline were prepared in a similar manner to that described above. Three New Zealand white rabbits, aged 9 weeks and weighing over 1.6 kg at beginning of the dose injection, were used in rabbit pyrogen test.

A total of three rabbits received injections of 10 mL/kg of the extraction vehicle and extracts of the PP tube in saline into a marginal ear vein. After injection, the rectal temperatures of the animals were recorded every 30 min for a period of 3 h. The response of each individual rabbit was defined as the difference between the basal temperature prior to injection and the highest temperature recorded after the injection.

If none of the rabbits showed a temperature increase of ≥ 0.5 °C over their basal temperature, or when the sum of the temperature increases in three rabbits did not exceed 3.3 °C, the tested serum was classified as pyrogen-free. When only one or two rabbits showed a temperature increase of ≥ 0.5 °C but the sum of the temperature increases in the three rabbits exceeded 3.3 °C, the test was repeated using five different rabbits. If no more than three of the eight rabbits tested showed an individual temperature increase of ≥ 0.5 °C or more and if the sum of the temperature increases in the eight rabbits did not exceed 3.3 °C, the batch tested was also classified as pyrogen-free.

#### Maximization sensitization test

The maximization sensitization test was conducted according to ISO 10993-10:2010(E): Tests for Irritation and skin sensitization-Guinea pig Maximization^18^. Female Guinea pig were provided by Agribrands Purina Korea Inc. (Pyeongtack, Republic of Korea). The PP tube extracts in saline and CSO were prepared in a similar manner to that described above. Thirty guinea pigs, aged 6 weeks and weighing 300–400 g at beginning of the dose injection, were used in the maximization sensitization test.

The intradermal induction phase of the study involved two main procedures: intradermal treatment and dermal exposure with the closed patch technique. The treatments were as follows:

- Test group:
  - 2 injections with 0.1 mL of Freund’s Complete Adjuvant (FCA) mixed with incubated extraction vehicles (saline or CSO) in a 1:1 volume/volume (v/v) ratio
  - 2 injections with 0.1 mL extracts of PP tube at 100% concentration
  - 2 injections with 0.1 mL of 50% v/v formulation of the 100% extracts in a 1:1 v/v ratio mixture of FCA and incubated extraction vehicles

- Negative group:
  - 2 injections with 0.1 mL of FCA mixed with incubated extraction vehicles in a 1:1 v/v ratio
  - 2 injections with 0.1 mL of incubated extraction vehicles
  - 2 injections with 0.1 mL of 50% v/v formulation of the incubated extraction vehicles in a 1:1 v/v ratio mixture of FCA and incubated extraction vehicles

- Positive group:
  - 2 injections with 0.1 mL of FCA mixed with incubated extraction vehicles in a 1:1 v/v ratio
  - 2 injections with 0.1 mL of 1-chloro-2,4-dinitrobenzen (DNCB) incubated ethanol
  - 2 injections with 0.1 mL of 50% v/v formulation of DNCB in a 1:1 v/v ratio mixture of FCA and ethanol

In the topical induction, the dorsal surface hair of the guinea pig was shaved before conducting the closed patch. These animals were patched with 2 × 4 cm gauze, which had been soaked in the solutions of the test, negative, and positive groups. The patches remained in place for a duration of 48 h. In the subsequent trigger phase, the hair on left and right sides of the lateral abdomen was shaved before conducting the closed patches. These animals were patched with 2 × 2 cm gauze, which saturated with solutions specific to the test (left), negative (right), and positive (right) groups. These patches wre secured for a period of 24 h.

General symptoms were daily assessed, while body weight measurements were conducted on a weekly basis and before the animals were sacrificed. The responses to patch test were evaluated following the Magnusson and Kligman scale (Supplementary Table 4) at 24 and 48 h after the end of the trigger phase. The sensitization rate was calculated by determining the ratio of animals showed a positive response to the total number of animals teasted.

### Sub-chronic toxicity testing

The sub-chronic toxicity tests were conducted according to ISO 10993-11:2006(E): Tests for Systemic toxicity-Subchronic Systemic Toxicity and ISO 10993-6:2007(E): Test for local effects after implantation^21^. A total of forty Sprague Dawley (SD) rats (20 male and 20 female) were provided by Orient Bio Co. Ltd. (Seongnam, Republic of Korea) and used in sub-chronic toxicity tests, with an age of 8 weeks at the beginning of implantation.

Before implantation, 10 mm of PE micro medical tubing intramedic (NC, BD Biosciences, PE205) and 2 × 10 mm of PP tube were sterilized using ethylene oxide gas. The gluteal area’s fur was shaved, and the surface was disinfected using povidone‑iodine and 70% ethanol. A 2 cm incision was made in the gluteal area to expose the underlying muscle, and then the sterilized tubes were implanted into the left and right sides of gluteal muscle. Following implantation, the incision was sutured and disinfected using povidone-iodine.

#### General observation

Throughout the experiment, any abnormal symptoms, including death, were daily observed and documented for each individual animal. Body weight was monitored at the start of implantation, once a week, upon necropsy. The quantity of supplied food and water, along with the remaining quantities, were recorded weekly, and the differences were calculated and documented.

#### Urinalysis

At the 13-week after implantation, urinalysis was performed on 5 rats from each group. This was accomplished by collecting fresh urine over a span of 4 h, using a urine collection plate. The analysis was conducted using an automatic urine chemistry analyzer (CLINITEK Advantus, Siemens, USA) in conjunction with urine test strips (Multistix 10 SG, Siemens, USA). Urine volume was quantified by collecting urine continuously for a period of 24 h. The urinalysis included inspection of the appearance, volume, specific gravity, protein, glucose, pH, ketone body, urobilinogen, bilirubin, nitrite, occult blood, and leukocytes. The grading standard for urinalysis was as described in Supplementary Table 5.

#### Ophthalmic examination

The condition of the animal’s eyes was examined both before the start of the experiments and before their sacrifice. Throughout the observation period, no abnormal symptoms pertaining to the eyeball were detected, thus obviating the need for a fundus examination. The evaluation of ocular abnormalities was conducted using a fundus camera (Genesis, Kowa Co. Ltd., Japan).

#### Hematology and blood/serum biochemistry

Animals were fasted overnight (16–24 h) while retaining access to water before blood collection. Following sacrifice, blood samples were obtained from the abdominal aorta, with the use of EDTA-2 K as an anticoagulant to prevent coagulation.

Hematology parameters were analyzed using a hematology analyzer (ADVIA 2120, Siemens, USA). The parameters included white blood cell count (WBC), red blood cell count (RBC), hemoglobin concentration (HGB), hematocrit (HCT), mean corpuscular volume (MCV), mean corpuscular hemoglobin (MCH), mean corpuscular hemoglobin concentration (MCHC), red blood cell distribution width (RDW), platelets (PLT), mean platelet volume (MPV), and reticulocyte (Retic).

A comprehensive panel of biochemistry parameters, including aspartate aminotransferase (AST), alanine aminotransferase (ALT), alkaline phosphatase (ALP), blood urea nitrogen (BUN), creatinine kinase (CRE), glucose (GLU), total cholesterol (CHO), total protein (TP), creatine phosphokinase (CPK), albumin (ALB), total bilirubin (T-BIL), triglycerides (TG), calcium (Ca), inorganic phosphorus (IP), chloride (Cl), sodium (Na), potassium (K), lactate dehydrogenase (LDH), magnesium (Mg), uric acid (UA), and albumin/globulin ratio (A/G), were measured using an automatic biochemistry analyzer (Hitachi Clinical Analyzer 7180, Hitachi, Japan). Plasma was isolated for determining aggregation time prothrombin time (PT) and activated partial thromboplastin time (APTT), which were quantified using a coagulation analyzer (ACL7000, Beckman, Germany).

#### Organ weight measurement

During the autopsy, all animals were weighed the following organs using an electronic scale, and the relative weight of each organ in relation to the body weight was calculated. The organs that were measured, included ovary (female), uterus (female), adrenal gland, pituitary gland, thymus, testis (male), epididymis (male), spleen, kidney, heart, lung, brain, and liver. Paired organs individually were measured.

#### Preservation of tissues and organs

Organs from all animals and grossly abnormal organs identified during the autopsy were excised. With the exception of the eyes (preserved in Davidson’s solution), testis and epididymis (preserved in Bouin’s solution), all organs were fixed in 10% neutral buffered formalin solution.

#### Histopathological examination

Histopathological examination were performed on all organs. The major organs were surgically excised and fixed in 10% buffered formalin (pH 7.4). After fixation, the tissue samples were dehydrated in a graded series of ethanol (70–99.9%), washed in toluene, and then embedded in paraffin. Thin tissue sections were obtained using a rotary microtome, and the material was stained with hematoxylin–eosin (H&E). Following staining, the sections were then examined under a microscope for pathological examinations, according to Supplementary Table 6 and 7.

### Statistical analysis

All quantitative data are presented as the mean ± S.D. of at least three independent experiments. Data analysis was performed separately for male and female groups. As the data followed a normal distribution, parametric tests were used to analyze these data. The statistical significance of the differences in sub-chronic toxicity observed between groups was assessed by independent-sample t-test. For in vitro cytotoxicity analysis, parametric analysis was conducted using one-way Analysis of variance (ANOVA), followed by Duncan's multiple range test, to assess the statistical significance of differences among the groups. Acute systemic toxicity and intradermal reactivity test were assessed using ANOVA, and multiple comparisons were performed with Dunnett’s test to identify mean values significantly different from the control groups. All statistical analyses were performed using the SPSS Statistics 12.0 software (SPSS, Inc., Chicago, IL, USA). Each *p*-value character was considered to indicate a statistically significant difference.

### Ethical approval

The study was approved by the Animal Care and Use Committee of KCL, Republic of Korea. All experiments were performed under the supervision of the the Animal Care and Use Committee of KCL, Republic of Korea in accordance with the relevant guidelines (IACUC-CU-00029 and CU18-00407-M1, Animal Care and Use Committee of KCL). All methods are reported in accordance with ARRIVE guidelines.

## Result

### Biocompatibility testing

#### In vitro cytotoxicity test

**Figure 1:**
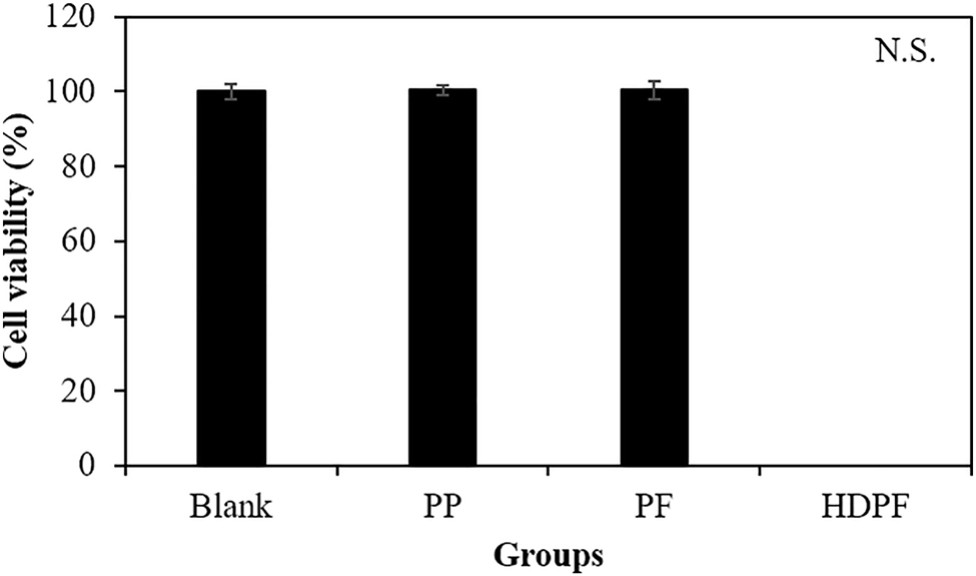
In vitro cytotoxicity of extracts of PE tube, PF, and HDPF for 48 h on L929 mouse fibroblast. N.S. Non-significant.

Firstly, we evaluated the in vitro cytotoxicity of extracts from PP tube, PF, and HDPF using viable cell counting assay. L929 cells were exposed to various extracts from all groups for 48 h. As shown in Fig. 1, exposure to extracts from both the PP and PF groups did not shown cytotoxicity. Whereas, cells incubated with extracts from HDPF group exhibited complete cell death (0% viability). Based on these results, we concluded that extracts from PP tube did not exhibit cytotoxicity on L929 cells.

#### Acute systemic toxicity study

**Figure 2:**
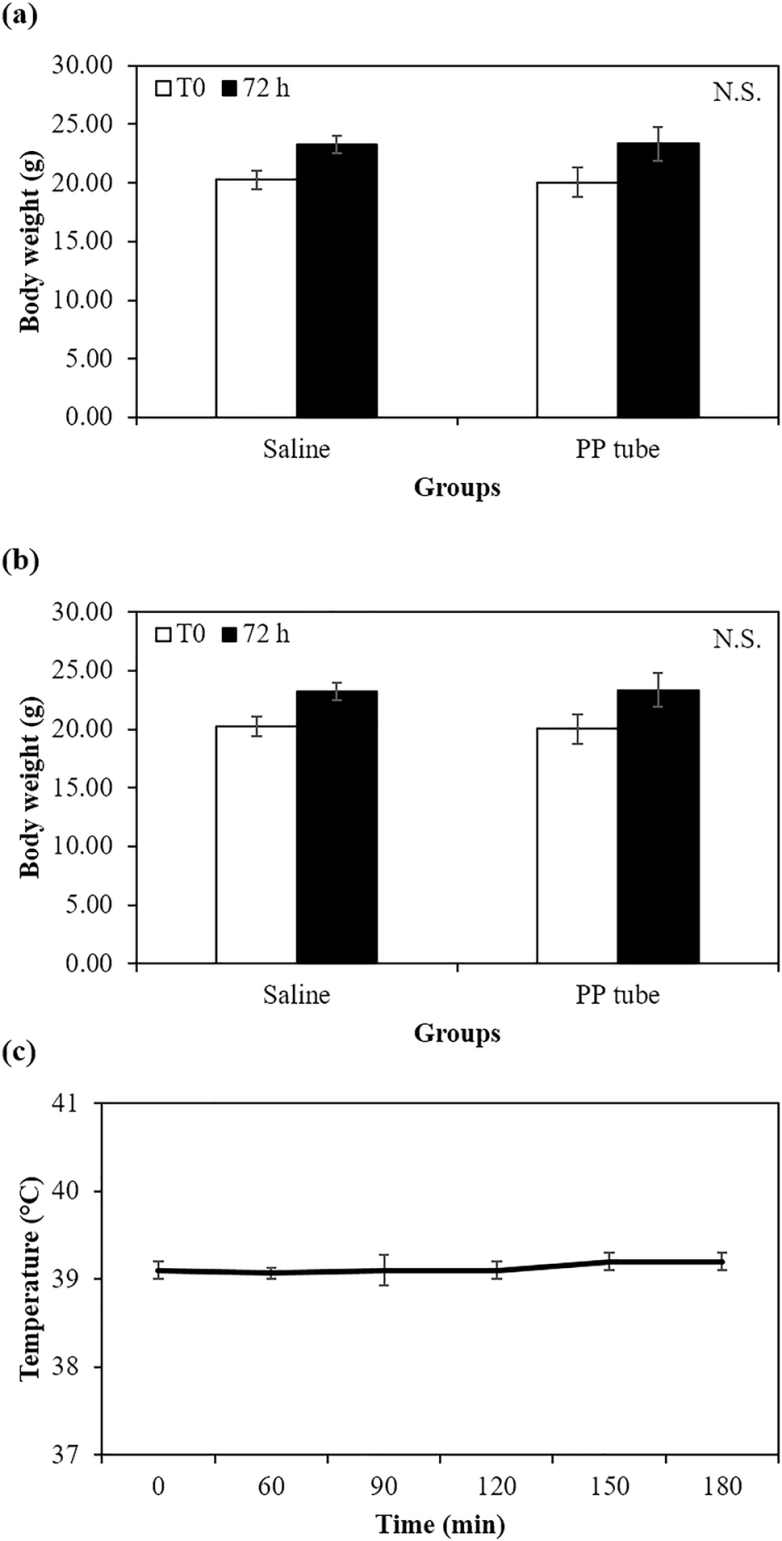
Effect on body weight change of injection of extracts from PP tube in (**a**) saline and (**b**) CSO in female mouse for a period of 3 days to evaluate acute systemic toxicity. (**c**) Temperature changes in rabbits following injection (10 mL/kg) of extract from PP tube in saline to evaluate pyrogenicity effect. N.S. Non-significant.

Subsequently, we evaluated the acute systemic toxicity of extracts from PP tube in either saline or CSO on ICR mice according to ISO 10993-11:2017(E): Tests for systemic toxicity-Acute systemic toxicity. There were no statistically significant differences in the body weight measured after 3 days from injection compared to those of the control group injected with saline or CSO (Fig. 2a,b and Table 1). Furthermore, all animals exhibited normal behavior, with no indication of abnormalities detected among any of the animals.

**Table 1:** Effect on body weight change of injection of saline and PE tube extract in female mouse for a period of 3 days to evaluate acute systemic toxicity in ICR mouse (n = 5, respective).

Group	Extraction route^a^	T0	72 h	Dead/Tested
Control	Saline^b^	20.25 ± 0.810	23.25 ± 0.725	0/5
	CSO^c^	20.14 ± 2.003	22.91 ± 2.391	0/5
PP tube	Saline^b^	20.04 ± 1.253	23.35 ± 1.461	0/5
	CSO^c^	20.06 ± 1.746	22.82 ± 2.105	0/5

^a^The dose of the extract was 50 mL/kg.

^b^Animals injected via the intravenous injection (I.V.) route.

^c^Animals injected via the intraperitoneal injection (I.P.) route; CSO: cottonseed oil.

#### Intradermal reactivity test

No alternations in body weight were measured among any of the animals throughout the daily observations for 3 days (Data not shown). Table 2 summarizes an overview of the scores for erythema, eschar formation, and edema, across the four groups. No significant reactions were observed in either of the animals injected with the extraction vehicle or extracts at any point during the study, except for very slight erythema and edema observed in some cases. This result indicates that the PP tube showed no intradermal reactivity in mice.

**Table 2:** Intradermal reactivity test in the rabbit: mean score for erythema and eschar formation and edema (n = 3, respectively).

Group	Extraction route	Scores^a^			
		T = 0	T = 24 h	T = 48 h	T = 72 h
Control	Saline^b^	0	0	0	0
	CSO^c^	0	0.13 ± 0.119	0.02 ± 0.034	0.04 ± 0.034
PP tube	Saline^b^	0	0	0	0
	CSO^c^	0	0.12 ± 0.107	0	0.02 ± 0.034
The overall mean score for polar control		0			
The overall mean score for polar extract of PP tube		0			
The overall mean score for non-polar control		0.4			
The overall mean score for non-polar extract of PP tube		0.3			

^a^Figures indicated the sum of scores for erythema and eschar formation and edema on fifteen sties divided by 15.

^b^Polar solvent.

^c^Non-polar solvent.

#### Pyrogen test

To evaluate the potential pyrogenic effect of the PP tube, the body temperature of guinea pigs was measured and recorded from 30 min prior to injection of the extracts of PP tube in saline, up to 3 h following injection at 30 min intervals. The differences between the baseline body temperature and the maximum body temperature recorded post-injection were calculated as the response. As shown in Fig. 2c, none of the three rabbits exhibited an increase in body temperature change exceeding a 0.5 °C compared to the body temperature of control groups. This result indicated that extracts of the PP tube contained no pyrogenic substances.

#### Maximization sensitization test

**Figure 3:**
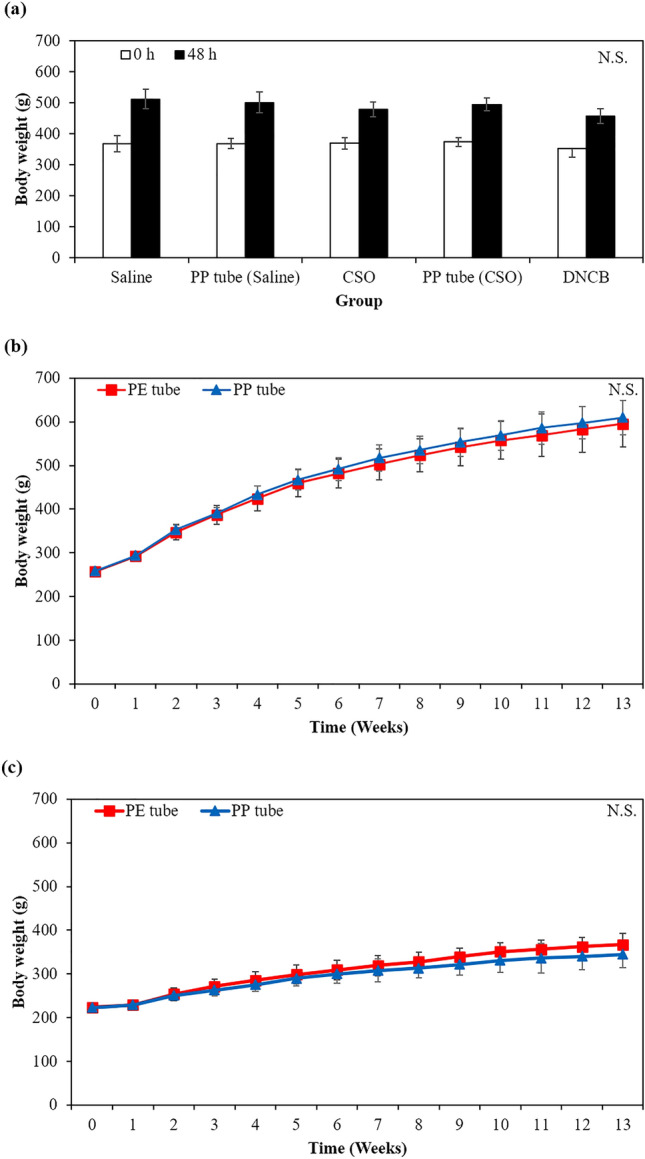
Effect on body weight of (**a**) female guinea pig after intracutaneous injection of extract from PP tube in saline and CSO and DNCB (in ethanol, negative control) in female mouse for a period of 48 h to evaluate maximization sensitization and rats after implantation on gluteal muscle of PE (N.C.) and PP tubes in (**b**) male and (**c**) female rats for a period of 13 weeks (n = 10, respective). (**b**). N.S. Non-significant.

In Fig. 3a, all experimental groups showed no significant differences in body weight measured 24 h after the beginning of the closed patch application compared to those of the extraction vehicle.

Table 3 summarizes the sensitization grade, sensitization rate, and number of animals exhibiting a sensitization response after 24 and 48 h of dermal application in guinea pigs. No sensitization was observed in the animals subjected to the application of either the extraction vehicle or extracts of the PP tube, except in the PC group. Remarkably, no fatalities were recorded among the experimental animals (Supplementary Table 8).

**Table 3:** Signs of toxicity and skin response score after intradermal injection of extract from PP tube in saline and cottonseed oil (CSO) and DNCB in female guinea pigs for a period of 48 h.

Group	Extraction route	Signs of toxicity^a^	T = 24 h			T = 48 h		
			Grade	Number of response	Sensitization rate (%)	Grade	Number of response	Sensitization rate (%)
Control	Saline^b^	None	0	0/5	0	0	0/5	0
	CSO^c^	None	0	0/5	0	0	0/5	0
PP tube	Saline^b^	None	0	0/10	0	0	0/10	0
	CSO^c^	None	0	0/10	0	0	0/10	0
DNCB	Ethanol	None	2.2 ± 0.84	5/5	100	2.0 ± 1.00	5/5	100

^a^Summary of clinical observations—Day 0 through Day 23.

^b^Polar solvent.

^c^Non-polar solvent.

### Sub-chronic toxicity testing

#### Clinical observations and survival

No mortalities were observed in SD rats throughout the 13-week period of implantation of PE micro medical tubing intramedic (PE group, NC) and PP tube in the gluteal muscle. None of the rats (both male and female) exhibited any obvious abnormal clinical signs of toxicity. Specifically, there were no discernible alternations in the skin and fur, eyes, respiratory rate, autonomic response (such as salivation, perspiration, and piloerection), or stereotyped activities observed at the beginning and the entire period of the in vivo sub-chronic toxicity study. Notable, no clinical indications of toxicity were observed within for the PE and PP group. Any slight changes or behaviors noted in SD rats during the study period were considered typical observations for this strain.

#### Effect on body weight, food intake, and water consumption

**Figure 4:**
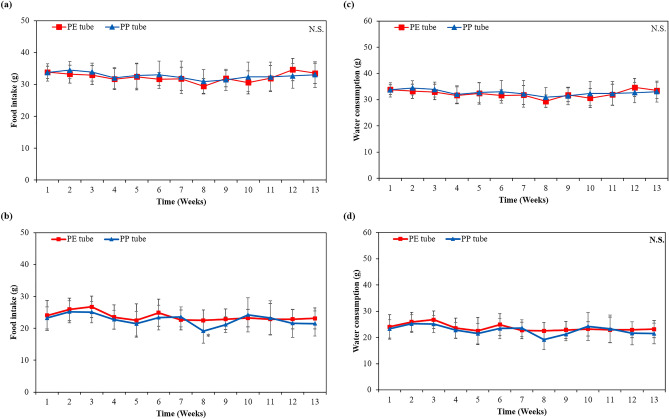
Effect on (**a**,**b**) food intake and (**c**,**d**) water consumption after implantation on gluteal muscle of the PE (negative control) and PP tubes in (**a**,**c**) male and (**b**,**d**) female rats for a period of 13 weeks of the sub-chronic toxicity study (n = 10, respective). ^*^p < 0.05 indicates significant difference compared to negative control group. N.S. Non-significant.

Supplementary Table 9 and Fig. 3b,c provide the body weight dataset for the PE and PP groups. The body weight of all animals steadily increased over 13-week period, and there was no significant difference between both groups, regardless of sex. Additionally, there was no significant difference in final body weight gained during 13 weeks between both groups and sexes. As a result, there was no indication of systemic toxicity or mortality after implantation. As shown in Fig. 4, both male and female animals showed no significant changes in food intake and water consumption within the PP group compared to the PE group (*p* > 0.05).

#### Urinalysis

The result of urinalysis in SD rats are summarized in Table 4. For both males and females, there was no significant difference (*p* > 0.05) in the urine analysis parameter (urine volume, glucose, bilirubin, ketone, specific gravity, occult blood, pH, protein, urobilinogen, leukocyte, and color of urine) between the PE and PP groups, despite a minor elevation in urine volume and specific gravity, and slight reductions in ketone, protein, and leukocyte levels.

**Table 4:** Urinalysis after implantation on gluteal muscle of the PE and PP tubes in male and female SD rats for a period of 13 weeks (n = 5, respective).

Group	PE tube		PP tube	
	Male	Female	Male	Female
Volume (mL)	13.00 ± 2.236	12.00 ± 4.528	14.20 ± 3.194	12.80 ± 1.304
Glucose	0	0	0	0
Bilirubin	0	0	0	0
Ketone	1.80 ± 0.447	1.00 ± 1.000	0.60 ± 0.548	0.20 ± 0.447
Specific gravity	3.00 ± 1.414	2.80 ± 0.837	3.80 ± 1.095	3.40 ± 1.517
Occult blood	0.20 ± 0.447	0.8 ± 0.447	0.4 ± 0.894	0
pH	8.50	8.40 ± 0.224	8.3 ± 0.447	8.20 ± 0.671
Protein	2.20 ± 0.837	1.20 ± 0.837	1.40 ± 0.548	0.40 ± 0.894
Urobilinogen	1	1	1	1
Leukocyte	1.20 ± 0.837	0.60 ± 0.548	0	0
Color	1.20 ± 0.447	1	1	1

#### Hematology and biochemistry

Table 5 presents the effect on hematological values after a 13-weeks period from the implantation of PE and PP tubes in the gluteal muscle. Notably, a significant increase in APTT were observed in males implanted with PP tube compared to PE tube. However, this elevated value remains within the normal range. Hence, for both male and female animals, there were no significant differences in blood parameters (a total 20 parameters; WBC, NE, LY, MO, EO, BA, RBC, HGB, HCT, MCV, MCH, MCHC, RCW, PLT, MPV, LUP, LUC, retic, PT, and APTT) between the PE and PP groups. Moreover, these parameters remained within the normal range even after the 13-weeks implantation period.

**Table 5:** Effect on hematological values after implantation on gluteal muscle of the PE and PP tubes in male and female SD rats for a period of 13 weeks (n = 10, respective).

Group		PE tube		PP tube	
		Male	Female	Male	Female
WBC (K/μL)		7.44 ± 1.585	4.58 ± 1.205	6.86 ± 1.239	5.10 ± 1.315
NE	K/μL	1.27 ± 0.386	0.77 ± 0.358	1.28 ± 0.498	0.86 ± 0.293
	%	17.44 ± 5.503	16.66 ± 5.971	18.80 ± 5.828	16.98 ± 4.753
LY	K/μL	5.79 ± 1.420	3.57 ± 1.042	5.25 ± 1.168	4.02 ± 1.137
	%	77.50 ± 5.351	77.89 ± 6.433	76.37 ± 6.764	78.52 ± 5.224
MO	K/μL	0.23 ± 0.071	0.14 ± 0.084	0.19 ± 0.077	0.13 ± 0.056
	%	3.08 ± 0.707	2.92 ± 1.317	2.71 ± 0.916	2.41 ± 0.711
EO	K/μL	0.10 ± 0.043	0.08 ± 0.038	0.09 ± 0.024	0.08 ± 0.035
	%	1.32 ± 0.399	1.93 ± 0.963	1.328 ± 0.545	1.61 ± 0.861
BA	K/μL	0.003 ± 0.005	0	0.001 ± 0.003	0
	%	0.06 ± 0.052	0.01 ± 0.032	0.04 ± 0.052	0.04 ± 0.052
RBC (M/μL)		8.53 ± 0.428	7.76 ± 0.370	8.75 ± 0.247	7.85 ± 0.244
HGB (g/mL)		14.31 ± 0.719	14.05 ± 0.640	14.05 ± 0.640	14.45 ± 0.382
HCT (%)		44.82 ± 2.503	41.99 ± 2.011	41.99 ± 2.011	42.78 ± 1.201
MCV (fL)		52.97 ± 1.949	54.13 ± 1.013	54.13 ± 1.013	54.48 ± 1.037
MCH (pg)		17.16 ± 0.638	18.13 ± 0.309	17.08 ± 0.752	18.41 ± 0.551
MCHC (g/dL)		32.63 ± 0.558	33.50 ± 0.422	32.90 ± 0.488	33.81 ± 0.569
RCW (%)		12.94 ± 0.738	11.18 ± 0.450	12.57 ± 0.640	11.48 ± 0.506
PLT (K/μL)		1,013.30 ± 88.956	977.40 ± 98.919	956.60 ± 104.753	1,017.10 ± 56.353
MPV (fL)		5.03 ± 0.279	6.46 ± 0.574	5.29 ± 0.623	6.14 ± 0.232
LUP (%)		0.65 ± 0.295	0.55 ± 0.268	0.70 ± 0.564	0.44 ± 0.190
LUC (K/μL)		0.05 ± 0.026	0.03 ± 0.011	0.05 ± 0.038	0.02 ± 0.010
Retic (%)		2.49 ± 0.463	2.14 ± 0.457	2.29 ± 0.364	2.71 ± 1.223
PT (sec)		9.72 ± 0.731	8.40 ± 0.367	9.79 ± 0.497	8.63 ± 0.576
APTT (sec)		15.59 ± 0.669	14.46 ± 1.585	17.13 ± 1.918*	14.94 ± 1.519

*WBC* White blood cell, *NE* Neutrophils, *LY* Lymphocyte, *MO* Monocyte, *EO* Eosinophil, *BA* Basophil, *RBC* Red blood cell, *HGB* Hemoglobin, *HCT* Hematocrit, *MCH* Mean corpuscular hemoglobin, *MCHC* Mean corpuscular hemoglobin concentration, *RCW* Red cell distribution width, *PLT* Platelet, *MPV* Mean platelet volume, *LUP* Percent of large unstained cell, *LUC* Large unstained cell, *Retic* Reticulocyte, *PT* Prothrombin time, *APTT* Activated partial thromboplastin time.

*p < 0.05 indicated significant difference compared to PE tube group.

The effect on serum biochemical values (a total 21 parameters; ALB, ALP, Ca, LDH, CHO, UA, CRE, GLU, AST, ALT, TP, BUN, T-BIL, IP, TG, Mg, CPK, Na, K, Cl, and A/G) after implantation of PE and PP tubes in the gluteal muscle is presented in Table 6. A significant increase in ALB was observed in males implanted with PP tube compared to PE tube, and a significant decrease in ALT for males and TG for females was observed in the PP group. Despite these changes, the elevated or decreased values remain within the normal range. The rest of the parameters showed no significant difference between the PE and PP groups, except several changed parameters, and they all remained within normal ranges across all groups (Table 6).

**Table 6:** Effect on serum biochemical values after implantation on gluteal muscle of the PE and PP tubes in male and female SD rats for a period of 13 weeks (n = 10, respective).

Group	PE tube		PP tube	
	Male	Female	Male	Female
ALB (g/dL)	2.34 ± 0.158	3.23 ± 0.271	2.52 ± 0.175*	3.00 ± 0.216
ALP (IU/L)	192.10 ± 57.506	78.70 ± 20.056	188.70 ± 43.043	90.90 ± 21.026
Ca (mg/dL)	9.42 ± 0.262	9.76 ± 0.241	9.64 ± 0.263	9.52 ± 0.312
LDH (IU/L)	907.60 ± 406.121	925.80 ± 410.769	749.70 ± 417.331	896.60 ± 398.922
CHO (mg/dL)	79.90 ± 17.754	113.80 ± 8.967	84.90 ± 20.883	105.90 ± 23.662
UA (mg/dL)	0.98 ± 0.164	0.92 ± 0.148	0.79 ± 0.242	1.01 ± 0.328
CRE (mg/dL)	0.43 ± 0.034	0.50 ± 0.041	0.43 ± 0.050	0.49 ± 0.044
GLU (mg/dL)	169.60 ± 18.530	161.10 ± 34.138	176.00 ± 19.698	146.50 ± 18.026
AST (IU/L)	107.60 ± 21.706	159.10 ± 86.039	91.70 ± 13.183	136.20 ± 57.577
ALT (IU/L)	37.60 ± 10.865	53.80 ± 21.509	29.20 ± 4.638*	40.00 ± 26.491
TP (g/dL)	6.22 ± 0.301	7.14 ± 0.372	6.44 ± 0.360	6.86 ± 0.395
BUN (mg/dL)	15.03 ± 0.706	14.82 ± 2.039	14.39 ± 2.340	16.15 ± 1.675
T-BIL (mg/dL)	0.05 ± 0.011	0.09 ± 0.032	0.06 ± 0.021	0.11 ± 0.043
IP (mg/dL)	6.36 ± 0.334	5.02 ± 0.590	6.27 ± 0.389	5.20 ± 0.552
TG (mg/dL)	48.70 ± 16.680	58.10 ± 25.532	66.40 ± 39.503	36.40 ± 18.222*
Mg (mg/dL)	2.11 ± 0.088	2.30 ± 0.125	2.16 ± 0.117	2.39 ± 0.137
CPK (U/L)	523.80 ± 202.984	505.00 ± 212.067	425.30 ± 238.016	480.70 ± 261.443
Na (mM/L)	144.10 ± 1.197	142.00 ± 1.333	143.90 ± 0.994	142.20 ± 1.476
K (mM/L)	4.73 ± 0.206	4.14 ± 0.217	4.63 ± 0.200	4.05 ± 0.303
Cl (mM/L)	109.20 ± 1.229	107.30 ± 1.889	108.50 ± 0.707	107.30 ± 1.636
A/G ratio	0.60 ± 0.048	0.83 ± 0.061	0.64 ± 0.041	0.78 ± 0.050

*ALB* Albumin, *ALP* Alkaline phosphatase, *Ca* Calcium, *LDH* Lactate dehydrogenase, *CHO* Total cholesterol, *UA* Uric acid, *CRE* Creatinine, *GLU* Glucose, *AST* Aspartate aminotransferase, *ALT* Alanine aminotransferase, *TP* Total protein, *BUN* Blood urea nitrogen, *T-BIL* Total bilirubin, *IP* Inorganic phosphorus, *TG* Triglyceride, *Mg* Magnesium, *CPK* Creatine phosphokinase, *Na* Sodium, *K* Potassium, *Cl* Chloride, *A/G ratio* Albumin/Globulin ratio.

*p < 0.05 indicated significant difference compared to PE tube group.

#### Organ weight measurement

The absolute and relative organ weight of both male and female rats are detailed in Table 7. In male rats, there was a significant increase in the relative organ weight of the pituitary gland (Table 7), and the absolute organ weight of right epididymis and pituitary gland also showed a significant increase compared to the PE group. In female rats, the absolute organ weight of liver, left adrenal gland, and pituitary gland exhibited significant decrease in the PP group compared to the PE group. However, no significant difference were observed in other absolute and relative organ weight between the PE and PP groups. Despite significant changes in the absolute and relative organ weights, considering the results from hematological and blood biochemical analyses (Tables 5 and 6), no specific abnormal findings were identified in the PP group, which makes it difficult to attribute these changes to toxicity.

**Table 7:** Effect on relative organ weight after implantation on gluteal muscle of the PE and PP tubes in male and female rats for a period of 13 weeks of the subacute toxicity study (n = 10, respective).

Organ		PE tube				PP tube			
		Male		Female		Male		Female	
		Absolute (g)	Relative (%)	Absolute (g)	Relative (%)	Absolute (g)	Relative (%)	Absolute (g)	Relative (%)
Kidney	Left	1.75 ± 0.142	0.29 ± 0.055	1.09 ± 0.085	0.32 ± 0.032	1.82 ± 0.209	0.31 ± 0.020	1.01 ± 0.096	0.31 ± 0.022
	Right	1.80 ± 0.153	0.30 ± 0.058	1.11 ± 0.111	0.32 ± 0.040	1.80 ± 0.175	0.31 ± 0.018	1.02 ± 0.078	0.31 ± 0.019
Spleen		0.93 ± 0.139	0.17 ± 0.032	0.61 ± 0.050	0.18 ± 0.018	0.95 ± 0.116	0.16 ± 0.014	0.62 ± 0.093	0.19 ± 0.031
Liver		14.54 ± 1.739	2.56 ± 0.177	8.87 ± 0.917	2.55 ± 0.223	15.30 ± 2.236	2.63 ± 0.208	7.97 ± 0.783*	2.46 ± 0.171
Adrenal gland	Left	0.03 ± 0.004	0.006 ± 0.0009	0.04 ± 0.003	0.01 ± 0.001	0.03 ± 0.006	0.006 ± 0.0013	0.03 ± 0.007*	0.01 ± 0.003
	Right	0.03 ± 0.006	0.006 ± 0.0009	0.04 ± 0.005	0.01 ± 0.001	0.04 ± 0.006	0.006 ± 0.0012	0.04 ± 0.003	0.01 ± 0.002
Thymus		0.40 ± 0.063	0.07 ± 0.009	0.36 ± 0.082	0.10 ± 0.019	0.36 ± 0.075	0.06 ± 0.013	0.29 ± 0.082	0.09 ± 0.021
Heart		1.71 ± 0.179	0.30 ± 0.028	1.13 ± 0.127	0.33 ± 0.029	1.74 ± 0.115	0.30 ± 0.024	1.09 ± 0.113	0.34 ± 0.032
Lung		1.89 ± 0.137	0.33 ± 0.025	1.40 ± 0.075	0.40 ± 0.031	1.95 ± 0.182	0.34 ± 0.026	1.38 ± 0.130	0.43 ± 0.033
Brain		2.27 ± 0.087	0.40 ± 0.031	2.04 ± 0.072	0.59 ± 0.048	2.24 ± 0.068	0.39 ± 0.022	2.02 ± 0.129	0.63 ± 0.059
Pituitary gland		0.01 ± 0.001	0.002 ± 0.0002	0.02 ± 0.005	0.007 ± 0.0014	0.01 ± 0.002*	0.002 ± 0.0004*	0.02 ± 0.003*	0.006 ± 0.009
Testis	Left	1.95 ± 0.095	0.35 ± 0.045	–	–	2.01 ± 0.141	0.35 ± 0.027	–	-
	Right	1.93 ± 0.110	0.34 ± 0.046	–	–	2.01 ± 0.136	0.35 ± 0.029	–	-
Epididymis	Left	0.80 ± 0.060	0.14 ± 0.019	–	–	0.86 ± 0.069	0.15 ± 0.013	–	-
	Right	0.82 ± 0.058	0.14 ± 0.018	–	–	0.91 ± 0.080*	0.16 ± 0.015	–	-
Ovary	Left	–		0.04 ± 0.010	0.01 ± 0.003	–		0.05 ± 0.015	0.02 ± 0.004
	Right	–		0.04 ± 0.009	0.01 ± 0.003	–		0.05 ± 0.010	0.01 ± 0.003
Uterus		–		0.62 ± 0.070	0.18 ± 0.026		–	0.59 ± 0.135	0.18 ± 0.050

*p < 0.05 indicates significant difference compared to PE group.

#### Histopathological examination of organ and implantation sites

Histological examinations of the organs were conducted on the final day of implantation through macroscopic observation of tissue sections obtained from the PE and PP groups. The results of this examination are presented in Table 8. In some sections of both the PE and PP groups, local inflammatory cell infiltration was observed in organs, such as the prostate, heart, harderian gland, liver, pancreas, kidney, urinary bladder, and lung. Furthermore, several histopathological findings were observed, including mineralization and tubular dilatation in kidney, acinar atrophy in pancreas, mineralization in aorta and tongue, basophilic hypertrophy in salivary gland, and the presence of a cyst in femur.

**Table 8:** Effect on histopathological and microscopic findings after implantation on gluteal muscle of the PE and PP tubes in male and female SD rats for a period of 13 weeks (n = 10, respective).

Group		PE tube		PP tube	
		Male	Female	Male	Female
Liver	No microscopic findings	4	0	2	0
	Inflammation, mild	5	10	8	9
	Inflammation, moderate	1	0	0	1
	Fatty change	0	1	0	0
Kidney	No microscopic findings	8	8	6	6
	Inflammation, mild	2	0	4	3
	Tubular dilatation, mild	0	0	0	1
	Mineralization, mild	0	2	0	0
Adrenal gland	No microscopic findings	10	10	10	10
Urinary bladder	No microscopic findings	10	10	9	10
	Inflammatory cell infiltration, mild	0	0	1	0
Spleen	No microscopic findings	10	10	10	10
Pancreas	No microscopic findings	8	8	8	9
	Inflammation, minimal	2	1	1	1
	Inflammation, moderate	0	0	1	0
	Acinar atrophy, minimal	0	1	0	0
Thymus	No microscopic findings	10	10	10	10
Thyroid gland	No microscopic findings	10	10	10	10
Trachea	No microscopic findings	10	10	10	10
Esophagus	No microscopic findings	10	10	10	10
Tongue	No microscopic findings	10	9	10	10
	Mineralization	0	1	0	0
Lung	No microscopic findings	6	10	8	7
	Inflammatory cell infiltration, minimal	3	0	0	1
	Mineralization	1	0	2	2
Heart	No microscopic findings	8	10	9	10
	Cardiomyopathy	2	0	1	0
Maxillary lymph node	No microscopic findings	10	10	10	10
Mesenteric lymph node	No microscopic findings	10	10	10	10
Salivary gland	No microscopic findings	10	10	10	9
	Basophilic hypertrophy	0	0	0	1
Stomach	No microscopic findings	10	10	10	10
Small intestine	No microscopic findings	10	10	10	10
Large intestine	No microscopic findings	10	10	10	10
Skin	No microscopic findings	10	10	10	10
Eye	No microscopic findings	10	9	10	10
	Inflammatory cell infiltration, minimal	0	1	0	0
Brain	No microscopic findings	10	10	10	10
Pituitary gland	No microscopic findings	10	10	10	10
Femur	No microscopic findings	10	9	9	10
	Cyst	0	1	1	0
Spinal cord	No microscopic findings	10	10	10	10
Sciatic nerve	No microscopic findings	10	10	10	10
Sternum	No microscopic findings	10	10	10	10
Testis	No microscopic findings	10	10	10	10
Epidodymis	No microscopic findings	10	10	10	10
Prostate	No microscopic findings	6	–	–	-
	Inflammatory cell infiltration, mild	4	–	–	-
Seminal vesicle	No microscopic findings	10	–	–	-
Coagulation gland	No microscopic findings	10	–	–	-
Ovary	No microscopic findings	–	10	10	10
Uterus	No microscopic findings	–	10	10	10
Average value of observation findings		0	0	13.37	14.43

In the surrounding of implantation site, fibrosis, fatty infiltrate, angiogenesis, and inflammatory cell infiltration were observed in the PP groups. Based on scoring according to the irritant ranking score, the PP tube showed moderate irritation (Score: 9.0–15.0) in both male (13.37) and female (14.43) rats.

## Discussion

In the development of drugs and medical devices, it is essential to adhere to the standards set by the FAD and ISO for determining their toxicity^22^. Therefore, assessing the general safety of drugs and medical devices is crucial before their use in humans. The objective of this study was to evaluate the biocompatibility and sub-chronic toxicity of the PP tube, aiming to investigate its potential as an advanced endotracheal tube for preventing TS.

The result of the in vitro cytotoxicity test conducted on L929 cells indicated that the extracts from both the PP tube and PC had no adverse effect on cell viability, which is in stark contrast to the NC group, where 100% cell death was observed. Based on these promising results, we proceeded to further evaluate the biocompatibility and sub-chronic toxicity using in vivo models.

The body weight is a crucial parameter for assessing the overall health status of animals, with weight loss often serving as the earliest indicator of the adverse effects caused by drugs or medical devices^23,24^. A decrease in body weight exceeding 10% is recognized as a potential sigh of toxicity, even in the absence of other observable toxicity signs, and may be directly associated with a loss of appetite, indicating disorders in carbohydrate, protein, or fat metabolism^25,26^.

In both the acute toxicity, involving the injection of PP tube extracts and extraction vehicles into ICR mouse, and the sub-chronic toxicity, involving the implantation of PE and PP tubes into the gluteal muscle of SD rats, none of the animals exhibited significant changes in food and water intake patterns. Furthermore, the body weight of all animals progressively increased without any indication of abnormalities when compared to the PE groups. These findings suggest that the short- (3 days) and long-term (13 weeks) exposure to the PP tube did not affect the animal’s appetite, general growth, or lead to disorders in carbohydrate, protein or fat metabolism.

Moreover, we conducted additional tests to confirm the safety of the PP tube, including intradermal reactivity, maximization sensitization, and pyrogen tests. In the intradermal reactivity, the PP tube did not induce any irritation reaction, as indicated by the difference between the average score of PP group and the average score of control group being < 1.0 (0 and 0.04 in the polar and non-polar extraction vehicle group, respectively). This conforms to the normative guidelines ISO 10993–10 and is considered non-irritating. Furthermore, the maximization sensitization test demonstrated that the PP tube did not induce any allergic reactions, such as erythema, edema, and any adverse skin response, and no abnormal clinical observation were made. Additionally, the intradermal administration of the PP tube extract in saline to rabbits did not cause any significant increase in body temperature for 3 h. These results collectively suggest the favorable safety attributes of PP tube in terms of allergic reactions, maximization sensitization, and pyrogenicity.

The urinalysis results indicated that the PP tube did not induced any notable treatment-related adverse effects on urinalysis parameters in both male and female subjected. Although a rise in protein levels was observed in female rat, this change remained within the confines of the normal biological range.

Hematological analysis is important for evaluating the toxicity of drugs or medical devices, given the critical role of hematopoiesis and its susceptibility to the impact of toxic compounds^27,28^. In the sub-chronic toxicity study, all hematological parameters showed no significant differences, with the exception of APTT in male SD rat. Nevertheless, the elevated APTT value remains within the administration of PP tube extracts did not influence immune responses or the hematopoietic process.

Enzyme markers indicative of organ function, such as ALT, AST, ALP, and LDH, are important factors for estimating the toxicity of drugs and medical devices, given their capacity to reflect tissue damage resulting from chemical compounds^29,30^. Since the liver and kidneys are involved in the metabolism and detoxification of compounds and their metabolites, they represent prime target organs for toxic chemical compounds. Therefore, the assessment of liver and kidney functions in response to drugs or medical devices is a vital component of toxicity evaluation^31,32^. In this study, an elevation in ALB was observed in male rats implanted PP tube, while a reduction in ALT was observed in both male and female rats of PP group. These changed values fall within the normal range for SD rats, and other parameters showed non-significant differences when compared to the control group. These results indicate that the implantation of PP tube does not influence on the hepatic and renal functions of the animals.

Another key indicator of potential induced toxicity is the morphological changes of internal organs, including color, texture, and sizes, which can be observed through necropsy^33^. After sacrifice, it was observed that all internal organs within the PP group did not exhibit any differences in terms of color, texture, or enlargement, as compared to the control group. Absolute and relative organ weights are considered as one of the fundamental markers to evaluate the presence of organ damage in animals subsequent to treatment^25,34^. In the PP group, the organ weight were found to be similar to those of the control group, despite the occurrence of some significant changes, all of which remained within normal range.

Finally, histopathological evaluations were conducted on both the organs and the surrounding tissue of the implanted site, aimed at comprehending any changes in cellular structure. In the histopathological evaluation of the organs, some cellular changes were observed, including occurrences of inflammatory cell infiltration, mineralization, tubular dilatation, acinar atrophy, basophilic hypertrophy, and cyst, within specific animals. However, there was no evidence of cellular injury, inflammation, necrosis, or abnormal cellular arrangement observed in either of the groups.

Regarding the identified lesions within the organs, the differences between the control group and the PP group were minimal, and these lesions were localized in localized regions. Several lesions were detected in only a limited number of animals and exhibited sporadic patterns, seemingly unrelated to the implantation of the PP tube. This suggests that these lesions might be considered as background lesions, unrelated to the study treatment^35,36^. In the gross pathological examination of the surrounding tissue at the implantation site, no adverse changes such as hemorrhage, necrosis, pallor, infection and hematoma formation were observed. Furthermore, the histopathological evaluation of the implantation site suggested that the PP tube exhibited a level of moderate irritation.

## Conclusion

In the present study, the assessment of biocompatibility and sub-chronic cytotoxicity of PP tube, intended for the functional enhancement of the endotracheal tubes, were performed. These results revealed the absence of toxic and sensitization potential within the PP tube extracts, resulting in the classification of these extracts as non-toxic according to ISO regulations. Furthermore, it was confirmed that no sub-chronic toxicity, except moderate irritation, was observed following vivo implantation study for long-term (13-weeks). Based on these findings and considering the short duration (up to a maximum of 8 weeks) of tube intubation to trachea^37^, the PP tube would not be expected to induce adverse effects in humans when used for the treatment of TS.

### Supplementary Information

Supplementary Information.

## Data Availability

All data generated or analyzed during this study are included in this published article and its supplementary information files.
