# Supplementary material for: Biocompatibility and sub-chronic toxicity studies of phlorotannin/polycaprolactone coated trachea tube for advancing medical device applications
Source: Sci Rep. 2024 Feb 16;14:3945. doi: 10.1038/s41598-024-54684-8 (PMC10873353; doi:10.1038/s41598-024-54684-8)
Supplement: Supplementary file 1 — Supplementary Information. [file 41598_2024_54684_MOESM1_ESM.docx]

**Assessment of biocompatibility and sub-chronic toxicity studies of phlorotannin-polycaprolactone-coated trachea tube: An In-depth animal study for advancing medical device applications**

Tae-Hee Kim^a,b^ , Seong-Yeong Heo^c^, Gun-Woo Oh^d^, Won Sun Park^e^, Won-Kyo Jung^a,b,f*^

^a^ Research Center for Marine-Integrated Bionics Technology, Pukyong National University, Busan 48513, Republic of Korea; taehee94@pukyong.ac.kr (T.-H.K.); wkjung@pknu.ac.kr (W.-K.J.)

^b^ Marine integrated Biomedical Technology center, The National Key Research Institutes in Universities, Pukyong National University, Busan 48513, Republic of Korea

^c^ Jeju Bio Research Center, Korea Institute of Ocean Science and Technology (KIOST), Jeju 63349, Republic of Korea; syheo@kiost.ac.kr (S.-Y.H.)

^d^ National Marine Biodiversity Institute of Korea, Seochun, Chungcheongnam-do, 33662, Republic of Korea; ogwchobo@mabik.re.kr (G.-W.O.)

^e^ Department of Physiology, Kangwon National University School of Medicine, Chuncheon 24341, Republic of Korea; parkws@kangwon.ac.kr

^f^ Major of Biomedical Engineering, Division of Smart Healthcare, College of Information Technology and Convergence and New-senior Healthcare Innovation Center (BK21 Plus), Busan 48513, Republic of Korea

**- Supplementary materials -**

Supplementary Table 1. The number of animals used in our study

| **Experiment** | **Animal** | **Number** | **Sex** |
| --- | --- | --- | --- |
| **Acute systemic toxicity study** | ICR mouse | 20 | Female |
| **Intradermal reactivity test** | New Zealand white rabbit | 3 | Male |
| **Pyrogen test** | New Zealand white rabbit | 3 | Male |
| **Maximization sensitization test** | Guinea pig | 30 | Female |
| **Sub-chronic toxicity testing** | Sprague Dawley Rat | 20 | Male |
|  |  | 20 | Female |

Supplementary Table 2. Positive and negative control list and sample type for biocompatibility and sub-chronic toxicity test

| **Test** | **Sample type** | **Positive control/RM** | **Negative control/RM** |
| --- | --- | --- | --- |
| In vitro cytotoxicity | Extracts in MEM | 0.1% ZDEC polyurethane film | High-density polyethylene film |
| Acute systemic toxicity study | Extracts in saline or CSO | - | Extraction vehicle (Saline or CSO) |
| Intradermal reactivity test |  |  |  |
| Maximization sensitization test |  |  |  |
| Pyrogen test | Extracts in saline | - | Extraction vehicle (Saline) |
| Sub-chronic toxicity testing | Material | PE micro medical tubing intramedic | - |

Supplementary Table 3. Grading standard for assessment and scoring of erythema, eschar formation, and edema after injection of the control solution and eluate solutions of PP tube on dorsal skin in New Zealand White rabbit

| **Score** | **Erythema and eschar formation** | **Edema** |
| --- | --- | --- |
| 0 | No erythema formation | No edema |
| 1 | Very slight erythema (Barely perceptible) | Very slight edema (Barely perceptible) |
| 2 | Well-defined erythema | Well-defined edema  (Edges of area well-defined by definite raising) |
| 3 | Moderate-to-severe erythema | Moderated edema (raised approximately 1 mm) |
| 4 | Severe erythema (beet redness)  to slight eschar formation (injuries in depth) | Severe edema (raised > 1 mm and extending  beyond area of exposure) |

Supplementary Table 4. Grading standard for maximization test according to Magnusson and Kligman scale after closed patch with solutions of test, negative, and positive groups in female guinea pigs

| **Patch test reaction** | **Score** |
| --- | --- |
| No visible change | 0 |
| Discrete or patchy erythema | 1 |
| Moderate and confluent erythema | 2 |
| Intense Erythema and swelling | 3 |

Supplementary Table 5. Grading standard for urinalysis after implantation on gluteal muscle of the PE and PP tubes in male and female SD rats

| **Color** | | **Glucose, Bilirubin, Ketone, Occult blood, Protein, Robilinogen, Leukocyte** | | **Specific gravity** | | **Urobilinogen** | |
| --- | --- | --- | --- | --- | --- | --- | --- |
| **Color** | **Grade** | **Date** | **Grade** | **Date** | **Grade** | **Date** | **Grade** |
| Colorless | 0 | - | 0 | ≤ 1.005 | 1 | 0.2 | 1 |
| Straw | 1 | ± | 1 | 1.010 | 2 | 1.0 | 2 |
| Yellow | 2 | 1+ | 2 | 1.015 | 3 | 2.0 | 3 |
| Dark Yellow | 3 | 2+ | 3 | 1.020 | 4 | 3.0 | 4 |
|  |  | 3+ | 4 | 1.025 | 5 | 4.0 | 5 |
|  |  |  |  | ＞1.030 | 6 |  |  |

Supplementary Table 6. Grading standard for microscopic observation after implantation on gluteal muscle of the PE and PP tubes in male and female SD rats

| **Evaluation of encapsulation** | |
| --- | --- |
| **Capsule width** | **Score** |
| **None** | **0** |
| **≤ 0.5 mm** | **1** |
| **0.6 ~ 1.0 mm** | **2** |
| **1.1 ~ 2.0 mm** | **3** |
| **＞ 2.0 mm** | **4** |
| **Not present** | **NP** |

Supplementary Table 7. Grading standard for histological evaluation after implantation on gluteal muscle of the PE and PP tubes in male and female SD rats

| **Cell type/response** | **Score** | | | | |
| --- | --- | --- | --- | --- | --- |
|  | **0** | **1** | **2** | **3** | **4** |
| **Polymorphonuclear cells** | 0 | Rare, 1-5/phf | 5-10/phf | Heavy infiltrate | Packed |
| **Lymphocytes** | 0 | Rare, 1-5/phf | 5-10/phf | Heavy infiltrate | Packed |
| **Plasma cells** | 0 | Rare, 1-5/phf | 5-10/phf | Heavy infiltrate | Packed |
| **Macrophages** | 0 | Rare, 1-5/phf | 5-10/phf | Heavy infiltrate | Packed |
| **Giant cells** | 0 | Rare, 1-5/phf | 5-10/phf | Heavy infiltrate | Packed |
| **Necrosis** | 0 | Minimal | Mild | Moderate | Severe |
| **Neovascularization** | 0 | Minimal capillary proliferation | Groups of 4-7 capillaries with supporting fibroblastic structure | Broad band of capillaries with supporting structures | Extensive band of capillaries with supporting fibroblastic structures |
| **Fibrosis** | 0 | Narrow band | Moderately thick band | Thick band | Extensive band |
| **Fatty infiltrate** | 0 | Minimal amount of fat associated with fibrosis | Several layers of fat and fibrosis | Elongated and broad accumulation of fat cells about the implant site | Extensive fat completely surrounding the implant |

phf: per high powered (400X) field

Supplementary Table 8. Survival of after implantation on gluteal muscle of the PE and PP tubes in male and female SD rats for a period of 13 weeks (n=10, respectively)

| **Group**  **Week** | **PE tube** | | **PP tube** | |
| --- | --- | --- | --- | --- |
|  | **Male** | **Female** | **Male** | **Female** |
| **1** | 10 | 10 | 10 | 10 |
| **2** | 10 | 10 | 10 | 10 |
| **3** | 10 | 10 | 10 | 10 |
| **4** | 10 | 10 | 10 | 10 |
| **5** | 10 | 10 | 10 | 10 |
| **6** | 10 | 10 | 10 | 10 |
| **7** | 10 | 10 | 10 | 10 |
| **8** | 10 | 10 | 10 | 10 |
| **9** | 10 | 10 | 10 | 10 |
| **10** | 10 | 10 | 10 | 10 |
| **11** | 10 | 10 | 10 | 10 |
| **12** | 10 | 10 | 10 | 10 |
| **13** | 10 | 10 | 10 | 10 |

Supplementary Table 9. Effect on body weight change after implantation on gluteal muscle of the PE and PP tubes in male and female SD rats for a period of 13 weeks to evaluate sub-chronic toxicity (n=10, respectively)

| **Group** | | **Initial weight (g)** | **Final weight (g)** | **Weight gain (g)** |
| --- | --- | --- | --- | --- |
| **PE tube** | **Male** | 256.80 ± 7.167 | 595.57 ± 53.244 | 338.77 ± 30.206 |
|  | **Female** | 223.38 ± 9.099 | 366.79 ± 26.419 | 143.41 ± 17.759 |
| **PP tube** | **Male** | 258.56 ± 5.568 | 609.46 ± 40.059 | 351.20 ± 22.814 |
|  | **Female** | 223.11 ± 4.690 | 344.86 ± 30.664 | 121.75 ± 17.677 |


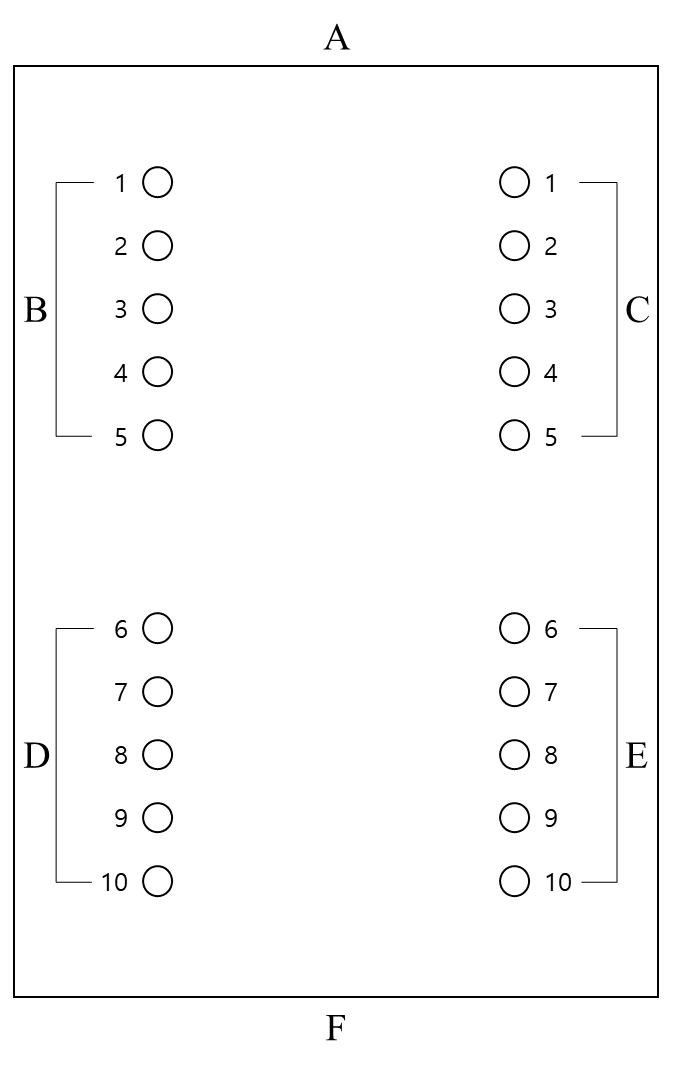


Supplementary Figure 1. Arrangement of injection sites for intradermal reactivity test on the New Zealand white rabbit. A: Cranial end, B: 0.2 mL injections of polar test extract (saline), C: 0.2 mL injections of polar extract of PE tube, D: 0.2 mL injections of non-polar test extract (cottonseed oil, CSO), E: 0.2 mL injections of non-polar extract of PE tube, F: Caudal end
